# Nedocromil sodium and diphenhydramine HCl ameliorate exercise‐induced arterial hypoxemia in highly trained athletes

**DOI:** 10.14814/phy2.15149

**Published:** 2022-01-10

**Authors:** Michael A. Coyle, Curtis S. Goss, Wesley J. Manz, Joel T. Greenshields, Robert F. Chapman, Joel M. Stager

**Affiliations:** ^1^ HH Morris Human Performance Laboratory, Department of Kinesiology, School of Public Health Indiana University Bloomington Indiana USA; ^2^ Department of Orthopaedic Surgery Emory University School of Medicine Atlanta Georgia USA; ^3^ Dr. Lawrence D. Rink Center for Sports Medicine and Technology Department of Intercollegiate Athletes Indiana University Bloomington Indiana USA

**Keywords:** blood‐gas barrier, gas exchange, histamine, interstitial pulmonary edema

## Abstract

**Introduction:**

Exercise‐induced arterial hypoxemia (EIAH) has been observed in highly trained endurance athletes during near maximal exercise, which may be influenced by a histamine‐mediated inflammatory response at the pulmonary capillary‐alveolar membrane. In order to test this hypothesis, we examined whether the mast cell stabilizer nedocromil sodium (NS) and H_1_‐receptor antagonist diphenhydramine HCL (DH) would ameliorate EIAH and mitigate the drop in arterial oxyhemoglobin saturation (S_a_O_2_) during intensive exercise.

**Methods:**

Seven highly trained male cross country runners (age, 21 ± 2 years; V̇O_2max_, 74.7 ± 3.5 ml·kg^−1^·min^−1^) participated in the study. All subjects completed a maximal exercise treadmill test to exhaustion, followed by three 5‐min constant‐load exercise bouts at 70%, 80%, and 90% V̇O_2max_. Prior to testing, subjects received either placebo (PL), NS, or DH.

**Results:**

Compared to PL, there was a significant treatment effect on S_a_O_2_ (*p* < 0.001) for both NS and DH during both constant‐load exercise and at V̇O_2max_. Post hoc tests revealed S_a_O_2_ values, compared to PL, were significantly higher at V̇O_2max_ and during DH trials and higher with NS at constant‐load intensities except at 70% (*p* = 0.13).

**Conclusion:**

The findings provide further evidence that histamine contributes directly or indirectly to the development of EIAH during intense exercise in highly trained athletes.

## Graphical abstract

The etiology of EIAH is currently unclear and likely idiopathic, and the theory that a histamine‐mediated inflammatory response may contribute to this phenomenon in some individuals merits further investigation. To our knowledge, the present study is the first to examine this theory in a group of athletes all with V̇O_2max_ values >70 ml·kg^−1^·min^−1^, while adding to the relatively sparse body of literature examining EIAH in cohorts of this training status. Our findings indicate that, at least in this cohort of highly trained runners with superior V̇O_2max_ values, pharmacological inhibition of the histamine response mitigated the decline in SaO2 without any change in ventilation, supporting previous literature which suggests that histaminergic release and action are related to the observed decrease in SaO2 during exercise.

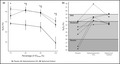

**New & Noteworthy**

The etiology of EIAH is currently unclear, and the theory that a histamine‐mediated inflammatory response may contribute to this phenomenon in some individuals merits further investigation. To our knowledge, the present study is the first to examine this theory in a group of athletes all with V̇O_2max_ values >70 ml·kg^−1^·min^−1^. Additionally, the present study adds to the relatively sparse body of literature examining EIAH in cohorts of this training status. Our findings indicate that, at least in this cohort of highly trained runners with superior V̇O_2max_ values, pharmacological inhibition of the histamine response mitigated the decline in S_a_O_2_ without any change in ventilation, supporting previous literature which suggests that histaminergic release and action are related to the observed decrease in S_a_O_2_ during exercise.

## 1 INTRODUCTION

Arterial oxyhemoglobin desaturation frequently observed during intensive normoxic exercise, termed exercise‐induced arterial hypoxemia (EIAH), is associated with a widened alveolar‐arterial oxygen difference (Dempsey et al., 1984; Prefaut et al., 2000). This phenomenon remains, to some extent, unclear in origin but has been well‐established among a variety of otherwise healthy populations of varying sexes, ages, and fitness levels (Dempsey & Wagner, 1999; Dominelli & Sheel, 2019; Prefaut et al., 2000). Inadequate hyperventilation, V̇_A_/Q̇_C_ inequality, veno‐arterial shunting, and diffusion limitation have been identified as the likely contributors to EIAH development (Dempsey & Wagner, 1999; Prefaut et al., 2000). The fundamental roles that inadequate hyperventilation and V̇_A_/Q̇C inequality play as underlying mechanisms of EIAH have been and continue to be investigated, and the role of shunts remains to be clarified (Dominelli & Sheel, 2019); however, the potential effects of a primary diffusion limitation on EIAH have recently received considerably less scientific attention.

Impaired gas exchange may occur secondary to injury and/or hemodynamic edema at the blood‐gas barrier (BGB) in the lung (Prefaut et al., 2000; West & Mathieu‐Costello, 1999). Various animal models have described mechanical stress failure of the BGB with increases in pulmonary arterial (and, consequently, pulmonary capillary) pressure (West & Mathieu‐Costello, 1999). In humans, there is considerable theoretical and experimental evidence indicating that intense physical exercise could conceivably elicit sufficient pressures to cause stress failure (Hopkins et al., 1997), or at least hemodynamic edema (McKenzie et al., 2005; Zavorsky, 2007). For example, Wagner et al. (Wagner et al., 1986) found pulmonary arterial pressure (PAP) was near 40 mmHg when healthy male subjects exercised at approximately 80%–90% of V̇O_2max_ at sea level, and similar results have been observed by others (Groves et al., 1987; Reeves et al., 1990). This could be exacerbated by the considerably larger Q̇_C_ values of trained athletes (Ekblom & Hermansen, 1968; Zhou et al., 2001), and it has been demonstrated that intense exercise can cause stress failure of the BGB in this population (Hopkins et al., 1997). Indeed, recent data demonstrated that, when corrected for body mass, maximal Q̇_C_, but not V̇O_2max_, was correlated with the decline in S_p_O_2_ from rest to maximal exercise (Schierbauer et al.,). These observations suggest that extravascular fluid may accumulate in the interstitium of the lung, at least transiently, enough so that decreases in S_a_O_2_ during exercise are evident until the pulmonary lymphatics can clear the interstitial space of the excess fluid post‐exercise (McKenzie et al., 2005). Furthermore, recent data showed that athletes with EIAH presented with higher exercising PAPs than non‐EIAH athletes, and that PAP was correlated with the decline in oxyhemoglobin saturation from rest to exercise (Durand et al., 2020).

It has been further theorized that mechanical stress at the BGB may induce an anaphylactoid response by which histamine release may worsen EIAH by contributing to increased vascular permeability, thus inducing permeability edema, and inflammation at the BGB (Prefaut et al., 2000). Histamine inhibition, therefore, may be a potential avenue by which to test the hypothesis of histaminic involvement and perhaps a means to alleviate EIAH. Indeed, histamine levels during exercise have been associated with reductions in arterial oxygen pressure (P_a_O_2_) (Anselme et al., 1994), while subsequent inhibition of histamine release results in a concomitant increase in S_a_O_2_ (Prefaut et al., 1997). Nedocromil sodium (NS), a mast cell stabilizer used in the treatment of asthmatics, attenuated EIAH in masters athletes (Prefaut et al., 1997). Likewise, diphenhydramine HCL (DH), an H_1_‐antihistamine, has been shown (albeit in sheep) to prevent both pulmonary edema and the increased lung vascular permeability due to histamine release (Brigham et al., 1976). While exercise is a well‐known initiator of systemic pro‐inflammatory mediator release, it is important to note that animal data have shown that the histamine‐forming capacity of the lung at rest and during exercise is considerably greater than that of other body compartments (Graham et al., 1964).

Thus, the purpose of this study was to investigate whether DH or NS could ameliorate EIAH (i.e., mitigate the decline in S_a_O_2_) in highly trained endurance runners with exceptional aerobic capacities, and therefore likely high Q̇_C_ values, to help illuminate the role of histamine in the development of EIAH. It was hypothesized that the decline in S_a_O_2_ during submaximal and maximal exercise could be mitigated by administration of DH and NS, two agents that suppress the action of histamine.

## 2 METHODS

### 2.1 Subjects

College‐aged National Collegiate Athletics Association (NCAA) Division 1 cross country athletes were recruited to participate in this study. The athletes competed in distance events ranging from the 1500‐m run to the marathon and were recruited based on their training background, an observed decline in arterial oxyhemoglobin saturation (S_a_O_2_) below 95% during heavy exercise (Dempsey & Wagner, 1999) and a V̇O_2max_ greater than 70 ml·kg^−1^·min^−1^ during previous study in our laboratory (Derchak et al., 2000), and no history of exercise‐induced asthma (EIA) or bronchoconstriction (EIB). All testing took place in the Indiana University Human Performance Laboratory in Bloomington, Indiana. The study was approved by the Indiana University Institutional Review Board for the protection of human subjects and all subjects gave written informed consent prior to participation in the study. Subject characteristics can be found in Table 1.

**TABLE 1:** Subject characteristics

	Mean ± SD (*N *= 7)
Age (year)	21.1 ± 2.0
Weight (kg)	66.7 ± 5.7
Height (cm)	177.4 ± 5.1
V̇O_2max_ (ml·kg^−1^·min^−1^)	74.7 ± 3.5

### 2.2 Experimental sequence

Subjects visited the lab on three occasions separated by at least 48 h but by no more than 1 week. Aside from receiving a placebo or different drug treatment prior to exercise, subjects performed identical work protocols on each visit to the laboratory. The protocol consisted of a progressive treadmill test to maximum exercise capacity, followed by three 5‐min constant‐load (McClaran et al., 1988) work bouts at 70%, 80%, and 90%, respectively, of their previously recorded V̇O_2max_ within the same testing session while S_a_O_2_ was continuously monitored via ear oximeter. Subjects received a 15‐min break between each work bout. Drug treatments were pseudo‐randomly assigned such that subjects received all drug treatments (PL, NS, and DH) by the end of the third visit to the laboratory. PL and NS were randomized in the first two visits, and DH was the last treatment for all subjects due to the nature of administration and the relatively longer half‐life (approximately 10 h in young adults Simons et al., 1990).

### 2.3 Drug delivery

In order to ensure mean peak plasma drug concentration during exercise, the following dosing schedule was observed: PL, isotonic saline (two puffs from an aerosol canister + nebulizer, GlaxoSmithKline) 15 min prior to initiation of testing procedures; NS, 4 mg (two puffs from an aerosol canister + nebulizer, Rhône‐Poulenc Rorer) 15 min prior to testing (Albazzaz et al., 1992); and DH, 25 mg (1 Benadryl^®^ KAPSEAL^®^ capsule, Pfizer‐Warner‐Lambert) was ingested orally 45 min prior to testing (Blyden et al., 1986).

### 2.4 Determination of V̇O_2max_

Upon completion of a 5‐min rest period, subjects walked for 2 min on a motor driven treadmill (model 18–60, Quinton, Bothell, WA) at 4.8 km·h^−1^ (3.0 mi·h^−1^) with 0% grade. After 2 min of walking, the speed of the treadmill was increased gradually to a speed chosen by the subject between 11.2 and 14.5 km·h^−1^ (7.0–9.0 mi·h^−1^). The speed of the treadmill remained constant throughout the test, and the same speed was used in each condition. After 2 min, and every 2 min thereafter, the grade of the treadmill was increased 2% until volitional exhaustion. The criteria used to assess V̇O_2max_ (Howley et al., 1995) included (1) a heart rate in excess of 90% of age‐predicted maximal heart rate (220–age), (2) a respiratory exchange ratio >1.10, and (3) a plateau (<150 ml·min^−1^) in V̇O_2_ despite a further increase in treadmill grade. In all the tests, at least two of these criteria were met. Expired ventilation (V̇_E_), oxygen uptake (V̇O_2_), and carbon dioxide output (V̇CO_2_) were continuously monitored via open‐circuit spirometry. Subjects breathed through a low‐resistance two‐way non‐rebreathing valve (model 2700, Hans Rudolph, St. Louis, MO), from which expired gases entered a 5‐L mixing chamber. Fractional expired concentrations of oxygen (F_E_O_2_) and carbon dioxide (F_E_CO_2_) were determined from a continuous sample of dried expired gas at a rate of 300 ml·min^−1^ using an Applied Electrochemistry S‐3A oxygen analyzer and CD‐3A carbon dioxide analyzer (Ametek, Thermox Instruments). The analyzers were calibrated with a gas of known composition in the physiologic range before and after each test. Fractional end‐tidal concentrations of oxygen (F_ET_O_2_) and carbon dioxide (F_ET_CO_2_) were simultaneously determined using a mass spectrometer (RAMS‐100; Marquette Medical Instruments). Inspired ventilation (V̇_I_) was measured using a thermistor flow meter (HEC 132C, Hector Engineering) calibrated at various flows against a Tissot spirometer. Analog signals from the analyzers, mass spectrometer, and the flow meter were continuously monitored and averaged over each minute of exercise with a data acquisition control system (Workbench for Windows v 2.2, Strawberry Tree). All pulmonary gas exchange calculations were performed by the data acquisition computer during the exercise test, providing numeric and visual outputs of V̇_E_ and V̇_I_ (1 min^−1^, BTPS) and V̇O_2_ and V̇CO_2_ (1 min^−1^, STPD), and RER. Heart rate was measured continuously via telemetry (model Vantage XL, Polar).

### 2.5 Constant‐load exercise

Subjects performed exercise at workloads approximately corresponding to 70%, 80%, and 90% V̇O_2max_, respectively. Once researchers observed a plateau in heart rate, V̇_E_, and V̇O_2_, subjects ran for 5 min. Metabolic and ventilatory variables were measured continuously throughout each workload as previous, as well as S_a_O_2_.

### 2.6 Arterial oxyhemoglobin saturation (S_a_O_2_) measurement

S_a_O_2_ was estimated using ear oximetry (Model 47201A; Hewlett‐Packard) due to the available personnel lacking requisite expertise to place an arterial catheter. Before each test, the oximeter was calibrated using an internal protocol according to the manufacturer's instructions. Prior to exercise, the subject's right ear pinna was cleaned and rubbed to increase blood flow. The ear oximeter was attached to a headgear, and an elastic bandage was used to further stabilize the oximeter.

The HP47201A is unique and, though no longer manufactured, accurately reflects arterial oxyhemoglobin saturation (Rebuck et al., 1983). This device uses eight wavelengths ranging from 650 to 1050 nm and an additional 18 molar extinction coefficients. In contrast, most current instruments use two or three wavelengths to determine oxy‐ and deoxyhemoglobin concentrations. The HP47201A oximeter outperforms forehead and finger pulse oximetry by estimating S_a_O_2_ in a manner that is satisfactorily independent of skin pigmentation, motion artifact, and other factors. Using advanced fiber optics and filters, the HP47201A has been reported to be accurate across a wide range of S_a_O_2_, with arterial S_a_O_2_ values registered at greater than 75% being underestimated by less than 2% (Smyth et al., 1986). Additionally, further calibration steps in our laboratory were performed as follows: A standard spectrophotometer cuvette was specially adapted in our laboratory to fit the fiber optic sample slit of the HP47201A oximeter transducer. Freshly drawn whole blood was evaluated via tonometry in order to produce a range of oxygen saturations. This blood sample was then introduced into the cuvette via glass syringes and measured by the ear oximeter transducer. One‐minute computer averages of S_a_O_2_ via the ear oximeter were referenced against the average of four samples analyzed by an OSM3 hemoximeter (Radiometer). In the range of 60%–95% S_a_O_2_, the two independent measures never differed by more than 1.9% (range 0.2%–1.9%) and were significantly correlated (*r *= 0.99) (Chapman et al., 1998). The ear oximeter was routinely checked versus blood samples were analyzed with an ABL3000 blood gas analyzer (Radiometer) with acceptable repeatability. For S_a_O_2_ ranging from 65% to 98%, the ear oximeter and blood gas analyzer were significantly correlated (*r *= 0.99), the average deviation across the range was <1.0%, and the maximum deviation at any point was 2.1%.

### 2.7 Statistical analysis

Prior to analysis, data were inspected for outliers and checked for normality using QQ‐norm and kernel density plots. In order to test for potential differences between drug treatments in the dependent variables of interest, linear mixed models were used. All models included random effects for subject to account for the repeated measures design. Constant‐load data were evaluated using a two‐way model (treatment: PL, NS, or DH × intensity: 70%, 80%, or 90%) and data at V̇O_2max_ were evaluated using a one‐way model (treatment). Where significant main effects (treatment or intensity) or interaction were observed, post hoc tests were completed using the Dunnett's method for multiple comparisons to compare PL against the experimental condition (NS or DH). All data are presented as mean ± 1 SD unless stated otherwise. A priori statistical significance was set to *p *< 0.05. All statistical analyses were performed using R version 4.0.1 (R Foundation for Statistical Computing).

## 3 RESULTS

### 3.1 Constant‐load exercise

**FIGURE 1:**
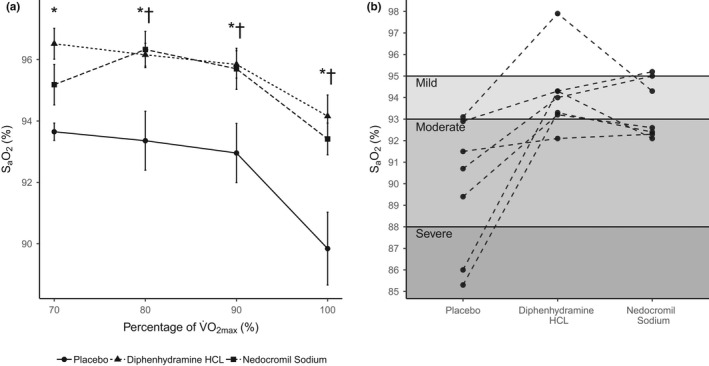
S_a_O_2_ for the submaximal constant‐load exercise at 70%, 80%, and 90% of V̇O_2max_ by group (left) and at V̇O_2max_ by subject (right) after ingestion of NS, DH, and PL

Average S_a_O_2_ data in the PL, NS, and DH treatment conditions are presented in Figure 1a, all other variables are presented in Table 2. Results showed a treatment effect in S_a_O_2_ (*p *< 0.001), with DH being greater than PL at 70% (2.72%, CI 1.11%–4.33%, *p* = 0.003), 80% (2.80%, CI 1.30%–4.30*%*, *p *= 0.001), and 90% (2.89%, CI 1.39%–4.39%, *p *< 0.001), and NS being greater than PL at 80% (2.97%, CI 1.47%–4.47%, *p *< 0.001) and 90% (2.74%, CI 1.24%–4.24%, *p* = 0.002), but not at 70% (1.47%, CI −0.14% to 3.08%, *p* = 0.132). A significant treatment effect in tidal volume (*V*_t_) was observed (*p* = 0.016); however, it appears that the observed effect was due to a difference between DH and PL at 70% intensity (−0.18 L, CI −0.33 to −0.23 L, *p *= 0.042). All other variables showed non‐significant main effects for treatment during the constant‐load submaximal exercise. Significant main effects for intensity were observed for V̇_E_ (*p *< 0.001), *f*_B_ (*p* = 0.043), *V*_t_ (*p *< 0.001), VO_2_ (*p *< 0.001), VCO_2_ (*p *< 0.001), RER (*p* = 0.001), V̇_E_/V̇O_2_ (*p *< 0.001), V̇_E_/V̇CO_2_ (*p* = 0.002), and HR (*p* < 0.001), but not in SaO_2_ (*p *= 0.660). No interactions between intensity and treatment were observed for any of the variables (*p *≥ 0.10).

**TABLE 2:** Group data from the last 3 min of submaximal constant‐load exercise at 70%, 80%, and 90% of V̇O_2max_ after ingestion of NS, DH, and PL

	70% of V̇O_2max_			80% of V̇O_2max_			90% of V̇O_2max_		
Variable	PL	DH	NS	PL	DH	NS	PL	DH	NS
% V̇O_2max_	72.2 ± 5.2	70.6 ± 5.2	69.9 ± 6.7	80.0 ± 4.2	76.1 ± 5.2	78.4 ± 3.6	89.1 ± 3.8	84.5 ± 9.5	89.9 ± 5.5
V̇_E_ (L·min^−1^)	90.6 ± 13.3	90.7 ± 14.4	86.9 ± 14.1	102.8 ± 12.4	101.4 ± 13.8	102.5 ± 12.3	124.6 ± 15.4	122.9 ± 17.1	124.7 ± 17.7
*f*_B_ (br·min^−1^)	49.5 ± 15.9	52.3 ± 16.3	47.3 ± 14.4	48.9 ± 14.8	50.3 ± 14.2	48.6 ± 12.1	53.1 ± 12.8	52.6 ± 12.2	51.1 ± 9.8
*V*_t_ (L·min^−1^)	1.98 ± 0.61	1.80 ± 0.43*	1.93 ± 0.47	2.23 ± 0.52	2.09 ± 0.45	2.24 ± 0.43	2.44 ± 0.47	2.34 ± 0.46	2.46 ± 0.39
V̇O_2_ (L·min^−1^)	3.52 ± 0.47	3.47 ± 0.36	3.45 ± 0.4	3.93 ± 0.24	3.81 ± 0.36	3.9 ± 0.36	4.39 ± 0.42	4.23 ± 0.52	4.49 ± 0.6
V̇CO_2_ (L·min^−1^)	2.80 ± 0.34	2.75 ± 0.31	2.73 ± 0.37	3.37 ± 0.22	3.27 ± 0.31	3.43 ± 0.28	4.07 ± 0.57	3.89 ± 0.58	4.16 ± 0.61
RER	0.79 ± 0.02	0.79 ± 0.02	0.79 ± 0.04	0.86 ± 0.04	0.86 ± 0.04	0.87 ± 0.02	0.93 ± 0.06	0.92 ± 0.03	0.93 ± 0.04
V̇_E_/V̇O_2_	25.8 ± 2.2	26.1 ± 2.7	25.2 ± 2.6	26.3 ± 2.6	26.6 ± 2.2	26.2 ± 2.3	28.5 ± 1.9	29.0 ± 1.8	27.9 ± 1.8
V̇_E_/V̇CO_2_	32.6 ± 3.3	33.0 ± 3.6	31.9 ± 3.2	30.5 ± 4.0	31.0 ± 3.2	30.0 ± 2.5	30.8 ± 2.8	31.5 ± 2.5	30.2 ± 2.5
HR (bpm)	157.8 ± 6.4	154.8 ± 6.9	155.2 ± 7.4	165.6 ± 8.1	162.0 ± 10.7	163.4 ± 10.9	175.4 ± 10.6	172.9 ± 9.1	174.6 ± 11.4

Note

Key: %V̇O_2max_, true percentage of V̇O_2max_ elicited; V̇_E_, minute ventilation; *f*_B_, frequency of breathing; *V*_t_, tidal volume; V̇O_2_, oxygen uptake; V̇CO_2_, carbon dioxide production; RER, respiratory exchange ratio; HR, heart rate; *denotes *p *< 0.05 within intensity between conditions.

### 3.2 Maximal exercise

All subjects satisfied at least two criteria for V̇O_2max_ during progressive exercise. S_a_O_2_ data during V̇O_2max_ are presented in Figure 1B, including shaded regions representing the severity of EIAH (Dempsey & Wagner, 1999) (mild, 95%–93%; moderate, 88%–93%; and severe: <88%). All other data during V̇O_2max_ are summarized in Table 3. At V̇O_2max_, a significant treatment effect was observed in SaO_2_ (*p* = 0.004), with DH (4.31%, CI 1.96%–6.67%, *p *= 0.004) and NS (3.57%, CI 1.21%–5.93%, *p* = 0.012) measurements being greater than PL. No other treatment effects were observed for any of the remaining variables (*p *≥ 0.197).

**TABLE 3:** Group data at V̇O_2max_ after ingestion of NS, DH, and PL

Variable	PL	DH	NS
V̇_E_ (L·min^−1^)	169.7 ± 16.6	167.5 ± 19.8	167.0 ± 16.7
*f*_B_ (br·min^−1^)	58.6 ± 7.6	58.7 ± 9.2	59.6 ± 4.2
*V*_t_ (L·min^−1^)	2.9 ± 0.4	2.9 ± 0.4	2.8 ± 0.4
V̇O_2_ (L·min^−1^)	4.93 ± 0.49	5.01 ± 0.36	4.99 ± 0.54
V̇CO_2_ (L·min^−1^)	5.61 ± 0.65	5.47 ± 0.64	5.53 ± 0.65
RER	1.14 ± 0.08	1.09 ± 0.09	1.1 ± 0.04
V̇_E_/V̇O_2_	34.4 ± 2.1	33.3 ± 2.1	33.6 ± 2.6
V̇_E_/V̇CO_2_	30.4 ± 1.8	30.7 ± 2.1	30.4 ± 1.9
HR (bpm)	188.9 ± 6.4	185.6 ± 9.8	186.1 ± 8.8

Note

Key: V̇_E_, minute ventilation; *f*_B_, frequency of breathing; *V*_t_, tidal volume; V̇O_2_, oxygen uptake; V̇CO_2_, carbon dioxide production; RER, respiratory exchange ratio; HR, heart rate; * denotes *p *< 0.05 between conditions.

## 4 DISCUSSION

The purpose of the present study was to test the hypothesis that a mast cell stabilizer (NS) and a H_1_‐receptor antagonist (DH) would mitigate the decline in S_a_O_2_ observed in highly trained athletes and provide potential evidence of a possible inflammatory involvement in etiology of EIAH. The principle novel findings are that NS and DH appear to ameliorate EIAH in these trained runners at V̇O_2max_ as well as during intense submaximal exercise. Primary supporting evidence for these conclusions are the significant elevations in S_a_O_2_ at maximal and submaximal exercise workloads with ingestion of NS and DH when compared to PL, with the exception of NS at 70%. In neither drug treatment was a change in V̇O_2_ nor an increase in V̇_E_ evident. These findings suggest that a histamine‐mediated inflammatory response plays a role in the development of EIAH in this subset of highly trained endurance athletes with superior V̇O_2max_ values (i.e., >70 ml·kg^−1^·min^−1^).

Four primary, classic physiologic mechanisms theoretically responsible for *any* arterial oxyhemoglobin desaturation have been proposed: veno‐arterial shunt, inadequate hyperventilation, V̇_A_/Q̇_C_ mismatch, and diffusion limitation (Dempsey & Wagner, 1999; Prefaut et al., 2000). Our data suggest that in these highly physically trained athletes an inflammatory response initiating a primary diffusion limitation may play a role in the presentation of EIAH. Regarding the other three proposed mechanisms, the present study design was not sufficient to assess the contribution of intrapulmonary or intra‐cardiac shunts, but it is unclear how NS or DH would affect shunting if it were present. Furthermore, though inadequate hyperventilation has been frequently been theorized to contribute to EIAH at submaximal workloads (Dominelli & Sheel, 2019), and breathing low‐density helium–oxygen gas mixtures has been shown to increase ventilation at sea level by unloading the work of breathing on the lungs, partially attenuating EIAH in normoxia (Babb, 2001; Dempsey et al., 1984), we provide no evidence for this mechanism in the attenuation of EIAH in these highly fit subjects. As no significant differences in V̇_E_ were observed between experimental and control conditions at any workload, it seems unlikely the present cohorts’ improvements in S_a_O_2_ during the drug trials were associated with an increase in O_2_ availability. Furthermore, Prefaut and colleagues (Prefaut et al., 1997) observed a decrease in alveolar ventilation during exercise following administration of NS in a similar study. Finally, V̇_A_/Q̇_C_ inequality has been implicated as a major factor in the onset of EIAH, and partially attributed to disturbances to normal diffusive capacity at the pulmonary capillary (Schaffartzik et al., 1992). Therefore, we propose a set of circumstances present in at least this specific subject population (i.e., presumably healthy, highly trained endurance athletes with superior V̇O_2max_ values) by which primary diffusion limitation potentially leads to secondary V̇_A_/Q̇_C_ inequality causing moderate EIAH at both submaximal and maximal workloads.

Administration of NS and DH significantly improved the arterial oxyhemoglobin saturation of these athletes (Figure 1). These results indicate that histamine is, or at least can be, a mediator in the development of EIAH, similar to previous observations and conclusions in masters athletes while cycling (Prefaut et al., 1997). While the present cohort differs considerably from the Prefaut et al. (1997) report, our results complement their speculations specifically regarding NS. They suggest that “the increase in capillary transmural pressure in master athletes during maximal incremental exercise induces stress failure with endothelial breaks. This probably initiates interstitial fluid accumulation and inflammatory processes with histamine release, but this latter is canceled by nedocromil sodium.” (Prefaut et al., 1997). Because of the technical and safety constraints associated with doing so, neither Prefaut et al. (1997) nor the present study report direct evidence (i.e., increased lung capillary transmural pressure and/or interstitial fluid accumulation in the lung) for this hypothesis. Nevertheless, mitigating the action of histamine could plausibly reconcile both the V̇_A_/Q̇_C_ mismatch and the diffusion limitation observed in subjects who experience desaturation while performing heavy exercise (Hammond et al., 1986; Prefaut et al., 2000).

Previous work indicates that maximal (Hopkins et al., 1997) but not submaximal (Hopkins et al., 1998b) exercise may cause stress failure of the pulmonary capillaries in highly trained athletes. However, highly trained athletes appear to maintain relatively high PAP even during submaximal exercise (Hopkins, Gavin, et al., 1998), which may not cause outright stress failure but could still be an initiator of mast cell degranulation. The pharmacological agents used in the present study are known to inhibit the effects of histamine, though at different points in the inflammatory pathway: NS acts to stabilize mast cells hindering histamine release (Albazzaz et al., 1992), while DH acts as a competitive inhibitor to histamine (Church & Church, 2011). Exercise (especially intense exercise) is a well‐documented initiator of acute‐phase inflammatory processes, including stimulating the action of granulocytic cells (Camus et al., 1993; Suzuki et al., 2002). Indeed, various compounds that appear in greater concentrations in the blood of athletes who experience EIAH have been implicated in signaling and modulating histamine release from its progenitor cells (Mucci et al., 2000, 2001; Prefaut et al., 2000; Suzuki et al., 2002). Once released from basophils (blood derived) and mast cells (tissue derived), histamine is available to bind to the large quantity of H_1_ receptors available in the pulmonary circuit (Panula et al., 2015). The binding of histamine to pulmonary H_1_ receptors causes microvascular permeability edema and tissue inflammation characteristic of a number of pathological conditions (Brigham & Owen, 1975; Probst et al., 1978) which, even at subclinical levels not nearly considered anaphylaxis, would ostensibly impair gas exchange during exercise.

Even independent of histaminic action, it is conceivable that the summation of mechanical stresses on the human lung resulting in a transient, sub‐clinical pulmonary edema could impede normal diffusion capacities during maximal exercise (Hopkins et al., 1997; Naeije & Chesler, 2012). Recent data from Durand et al. (Durand et al., 2020) found that athletes with EIAH had higher PAPs than similarly trained athletes without EIAH, further supporting a hemodynamic initiating mechanism of EIAH in this cohort. Additionally, Schierbauer and colleagues found that, when corrected for body mass, maximal Q̇_C_, but not V̇O_2max_, was correlated with the decline in S_p_O_2_ from rest to maximal exercise (Schierbauer et al.,). It is possible that the highly trained subjects in our study approached maximal Q̇_C_ values meeting or, more likely, exceeding 30–35 L·min^−1^ (Table 1) (Zhou et al., 2001). Though there are recent data indicating a correlation between aerobic capacity and resting pulmonary capillary volume (V_C_) (Lalande et al., 2012), that study was performed on subjects with an average V̇O_2max_ value of 41.8 ml·kg·^−1^·min^−1^, and the only subject with a value exceeding 55 ml·kg^−1^·min^−1^ did not nearly have the highest pulmonary V_C_. It is otherwise generally accepted that the morphologic characteristics of the pulmonary vasculature do not change with exercise training, though human data are generally lacking (Laughlin et al., 2012). Thus, it is conceivable that aerobically trained athletes (as in the present study) may operate at the morphological limits of their pulmonary V_C_ to accept right ventricular outflow (La Gerche et al., 2011). Together, these extreme Q̇_C_ values pumping against an otherwise normal pulmonary vascular resistance would plausibly produce exceptional PAPs (Wagner et al., 1986) and pulmonary capillary wedge pressures (Hopkins, Gavin, et al., 1998), potentially inducing mechanical failure leading to at least a transient edema similar to that seen in animal models (Wagner et al., 1986; West & Mathieu‐Costello, 1999). This phenomenon would increase the effective thickness of the membrane, presumably impeding O_2_ diffusion capacity, leading to a low V̇_A_/Q̇_C_, and ultimately resulting in observable declines in S_a_O_2_. Stress failure of the pulmonary capillary may itself be a sufficient stimulus for histamine release and thus histamine concentrations might well be a marker of these factors rather than the initiating mechanism per se.

If superior aerobic fitness, and therefore a high Q̇_C_, is a prerequisite for young, healthy athletes to experience an inflammatory response leading to EIAH, then it stands to reason that anti‐inflammatory agents may not ameliorate EIAH in athletes with lower fitness capacities. Wetter et al. (Wetter et al., 2002) found that an anti‐inflammatory drug cocktail of 60 mg of fexofenadine (antihistamine), 600 mg of zileuton (leukotriene‐synthesis inhibitor), and 5.25 mg of nedocromil sodium did not improve S_a_O_2_ in nine male and eight female young athletes. They concluded that “airway inflammation is of insufficient magnitude to cause impairments in gas exchange” as the administered drugs did not alleviate EIAH, nor were increased inflammatory markers observed in post‐exercise sputum (Wetter et al., 2002). Additionally, Hodges et al. (2005) found that dosing twice daily over 7 days with a cocktail of 400 µg of salbutamol (selective short acting β_2_‐adrenergic antagonist bronchodilator) and 500 µg of fluticasone (an anti‐inflammatory corticosteroid) did not significantly affect S_a_O_2_ compared to placebo in nine male cyclists. However, male subjects in the Wetter (Wetter et al., 2002) and Hodges (Hodges et al., 2005) studies had average V̇O_2max_ values of <62 ml·kg^−1^·min^−1^, whereas our subjects had an average V̇O_2max_ of nearly 75 ml·kg^−1^·min^−1^, with no subjects below 70 ml·kg^−1^·min^−1^. If the onset of EIAH is mediated by the presence of an inflammatory response and a sub‐clinical, transient pulmonary edema, then individuals lacking abnormally high Q̇_C_’s––and subsequently lacking abnormally high PAP’s––would not be afflicted in the same manner as those who do. This inference garners additional support from the higher arterial oxyhemoglobin saturation values observed in Wetter (Wetter et al., 2002) and Hodges (Hodges et al., 2005) studies, which were approximately 92% S_a_O_2_ at max exercise in the placebo condition. This is in comparison to the present subjects’ desaturation in the placebo condition to 90% S_a_O_2_ at max, with two individuals reaching nearly 85% S_a_O_2_.

### 4.1 Limitations

The main limitation of the study is that histamine nor other markers of pulmonary inflammation were not measured, so it is not explicitly clear that an inflammatory response including transient sub‐clinical edema occurred. Nor can we explicitly confirm that this was consequently ameliorated by the anti‐inflammatory agents administered. However, it is unclear what other physiological process the pharmacological agents could have altered that would have caused the observed increase in S_a_O_2_ as the agents used in the present study are not known to alter the oxyhemoglobin dissociation relationship.

The study is additionally limited methodologically in two other facets. First, multiple inert gas elimination technique and arterial blood samples were not used, so the supposition that a diffusion limitation and subsequent V̇_A_/Q̇_C_ mismatch was the source of the declining S_a_O_2_ and was subsequently ameliorated by the medications is an argument of exclusion and solely supported by previous literature. That is, it is unclear what mechanisms the medications could have affected other than those theorized. Though an unchanged exercise ventilation does not exclude a change in alveolar ventilation, we do not believe that alveolar ventilation was changed in the medicated condition. If anything, tidal volume was reduced in the DH condition, suggesting more dead space ventilation and less alveolar ventilation. Furthermore, a similar study (Prefaut et al., 1997) found a *decrease* in alveolar ventilation in the medicated (nedocromil sodium) versus placebo condition, suggesting that increased alveolar ventilation is not a mechanism responsible for the observations in the present study.

Second, challenge or exercise tests were not used to determine the presence of EIA and EIB, only an examination of each athlete's medical history. Therefore, presence of EIA or EIB cannot be definitively excluded. It is important to note that Prefaut and colleagues (1997) observed similar results while definitively excluding EIA and EIB.

Additionally, as the current study measured neither nor PAP, the presence of transient pulmonary edema in elite‐level athletes similar to our subject cohort is supported solely by previous literature. Using a known regression equation (3), it is estimated that subjects in the present study with V̇O_2_ values between 4.2 and 5.6 l·min^−1^ maintained Q̇ between 28.3 and 36.7 L·min^−1^, respectively (Astrand et al., 1964). The resulting PAP would be considerable in this case, and it is conceivable that such stress could result in transient edema and minor injury at the BGB, ultimately causing an inflammatory response that is mitigated with the ingestion of pharmacological agents.

Participants and researchers were also not fully blinded to the drug condition and the DH was always the final drug condition, and this may have influenced the results of the study. All subjects had completed exercise tests in the lab prior to this study and were quite familiarized with the procedures, so it is unclear how the visit order or knowledge of the drug condition may have influenced cardiopulmonary variables during submaximal and maximal exercise tests.

Finally, only men were studied during this investigation, and therefore the results are not necessarily generalizable to similarly trained women. Access to this unique cohort was granted to us by only the men's university cross country coach, and women were unfortunately unavailable for inclusion. Data suggest that women more frequently present with EIAH than men (Dominelli & Sheel, 2019), so future studies examining the connection between histamine and EIAH should seek to include trained women.

## 5 CONCLUSION

Our data demonstrate that introduction of a histamine‐targeting, anti‐inflammatory agent (NS or DH) ameliorates EIAH in these highly trained runners. Due to methodological constraints with healthy fit subjects, our data do not provide direct evidence of the inflammatory responses associated with histamine and the lung vasculature. Indirectly, however, our data support the theory that these pharmacological agents relieved the transient edema and/or an inflammatory response caused by intensive exercise by suppressing the release (NS) or action (DH) of histamine, a compound that has been implicated in these mechanisms. EIAH observed in normoxia may be related to increased PAPs and changes in capillary permeability in concert with an inflammatory response within the lung. Future inquiries pertaining to the inflammatory process and the significance of histamine release during intensive exercise should explore the idiosyncratic etiology of EIAH in various populations and individuals with appropriate measures of cardiac performance, pulmonary pressures, and pulmonary interstitial fluid volumes.

## CONFLICT OF INTEREST

The authors do not declare any conflict of interest.

## AUTHOR CONTRIBUTIONS

MAC, RFC, and JMS designed the study. MAC recruited participants and collected the data. MAC, JTG, and CSG analyzed the data. JTG created figures and tables. All authors interpreted the data. MAC, CSG, WJM, and JTG wrote the manuscript. All authors edited the manuscript. All authors approved the final submission.
